# A high throughput array microhabitat platform reveals how light and nitrogen colimit the growth of algal cells

**DOI:** 10.1038/s41598-024-59041-3

**Published:** 2024-04-29

**Authors:** Fangchen Liu, Larissa Gaul, Andrea Giometto, Mingming Wu

**Affiliations:** 1 Department of Biological and Environmental Engineering, Cornell University, Ithaca, NY USA; 2 School of Civil and Environmental Engineering, Cornell University, Ithaca, NY USA

**Keywords:** Biophysics, Environmental sciences, Engineering

## Abstract

A mechanistic understanding of algal growth is essential for maintaining a sustainable environment in an era of climate change and population expansion. It is known that algal growth is tightly controlled by complex interactive physical and chemical conditions. Many mathematical models have been proposed to describe the relation of algal growth and environmental parameters, but experimental verification has been difficult due to the lack of tools to measure cell growth under precise physical and chemical conditions. As such, current models depend on the specific testing systems, and the fitted growth kinetic constants vary widely for the same organisms in the existing literature. Here, we present a microfluidic platform where both light intensity and nutrient gradients can be well controlled for algal cell growth studies. In particular, light shading is avoided, a common problem in macroscale assays. Our results revealed that light and nitrogen colimit the growth of algal cells, with each contributing a Monod growth kinetic term in a multiplicative model. We argue that the microfluidic platform can lead towards a general culture system independent algal growth model with systematic screening of many environmental parameters. Our work advances technology for algal cell growth studies and provides essential information for future bioreactor designs and ecological predictions.

## Introduction

Phytoplankton, including microalgae and cyanobacteria, are essential in maintaining a balanced ecosystem by supporting primary production and mediating the carbon cycle. Cyanobacteria, by producing and releasing oxygen as a byproduct of photosynthesis, contributed to the rise of oxygen on Earth over 2 billion years ago, and were essential for the evolution of life on Earth. Disruption of phytoplankton growth can lead to environmental problems such as harmful algal blooms (HABs)^1–3^, which deplete water resources and disrupt aquatic ecosystem function. HABs have been exacerbated by excessive nutrient inputs and climate change, including the effect of warming and increasing frequency and intensity of extreme storm, flooding and drought events^4–6^. In contrast to HABs, lipid production from controlled growth of microalgae is a promising avenue for clean alternative bioenergy^7–9^, and a number of microalgal species are excellent nutritional food sources for animals, including humans^10–12^. As such, a mechanistic understanding of the control and management of algal growth is an essential step towards finding solutions for sustainable aquatic ecosystems.

The biophysical (e.g. light and temperature) and biochemical (e.g. nitrogen and phosphorous) environment critically impacts the growth of microalgae. Traditionally, the environment has been considered as a major selective force acting at the gene level. Recent studies have revealed important roles played by the environment in the ecology, evolution, and development of biological organisms, within the so-called “eco-evo-devo” field^13–17^. Phenotypic plasticity of cells can emerge under time-varying environmental conditions, that help cells respond quickly to environmental fluctuations^18–20^. For example, microorganisms and the interactions among them can shape their environment, a phenomenon known as niche construction, leading to the co-evolution of organisms and their environment^21–24^. Despite the importance of cell-environment interactions in mediating algal growth, a mechanistic understanding of how complex environments regulate algal growth is limited. This is due to the lack of tools where environmental conditions can be directly controlled, while cell growth can be monitored in real-time and space.

Mathematical modeling of algal growth kinetics, especially in complex environmental conditions, is important for our basic understanding of algal growth in the natural environment, as well as bioreactor optimization and ecological prediction. Many algal growth models have been derived from first principles such as mass action kinetics and growth optimality. These models were critical in shaping our current understanding of algal growth dependence on multiple resources in natural waters as well as experiments with large-scale batch and continuous cultures^25–29^. Monod kinetics has been used successfully to describe algal growth under single nutrient or light intensity gradients^30^. Multiplicative Monod kinetics model has been proposed to describe algal growth under multiple physical and nutrient resources form^28,31–36^. However, verification of growth models is challenged by the lack of tools where both physical and nutrient parameters can be controlled precisely and are also high-throughput. Current macroscale algal cell culture systems, batch or chemostat bioreactors, are not designed to provide well defined control of light intensity due to light shading and fluid flows in the system. As a result, there exists a large number of different mathematical models proposed to describe algal growth in mixed nutrients or light intensity, and fitted parameters from prior experiments vary widely for the same micro-organism^29,37^. In this context, our ability to propose a unified model relies on the development of advanced engineering tools that can provide well defined complex environmental conditions for algal cell growth.

Microfluidics has emerged to provide well controlled environments for algal cell growth. The small size of the device allows for multiplexing (multiple light intensities and nutrient concentrations on one chip), thus obtaining large data set in one experiment. In particular, microfluidics allows to minimize the light shading problem that is intrinsic to macroscale systems. In addition, microfluidic devices are compatible with optical imaging, enabling quantitative studies of cells in both time and space. The miniaturized devices could also help lower the detection limit down to single cells and small populations, which overcomes the challenges of measuring bulk growth rates at very low nutrient concentrations or light intensities where the cultures are usually dilute. Using microfluidic platforms, algal growth has been studied extensively under single environmental gradients, including of nutrients^38–41^ and light^42,43^. These experiments established that algal cell growth dependence on single nutrient or light source can be described by Monod kinetics. Although dual chemical gradient generators have been reported to rapidly screen cellular (e.g. bacteria and animal cells) responses to multiple stressors^44–47^, few have been used to study synergistic effect of multiple nutrients on algal cell growth^48^. Photosynthesis is an important part of algal cell growth, and it has long been known that light and nitrogen could colimit algal growth. However, quantitative description of the colimitation has been limited to models parameterized with large scale cultures^29^. Thus, microfluidics, with its precise light and nitrogen control and multiplexing potential, offers the opportunity to carefully quantify their colimitation on algal growth^49,50^. We note that Nguyen et al. recently presented a millimeter-scale platform that examined the optimal light intensity and nutrient condition for lipid production of algal cells under controlled light and nutrient condition for cells immobilized in hydrogels^50^.

In this article, we present a microfluidic platform that can provide well defined nutrient and light gradients simultaneously for the growth of algal cells. Using this platform, we verified a mathematical model according to which nutrient and light colimit the growth of algal cells in the form of a multiplicative Monod kinetics.

## Results

### A microfluidic platform for growing photosynthetic microbes under a dual light and nitrogen gradient

**Figure 1:**
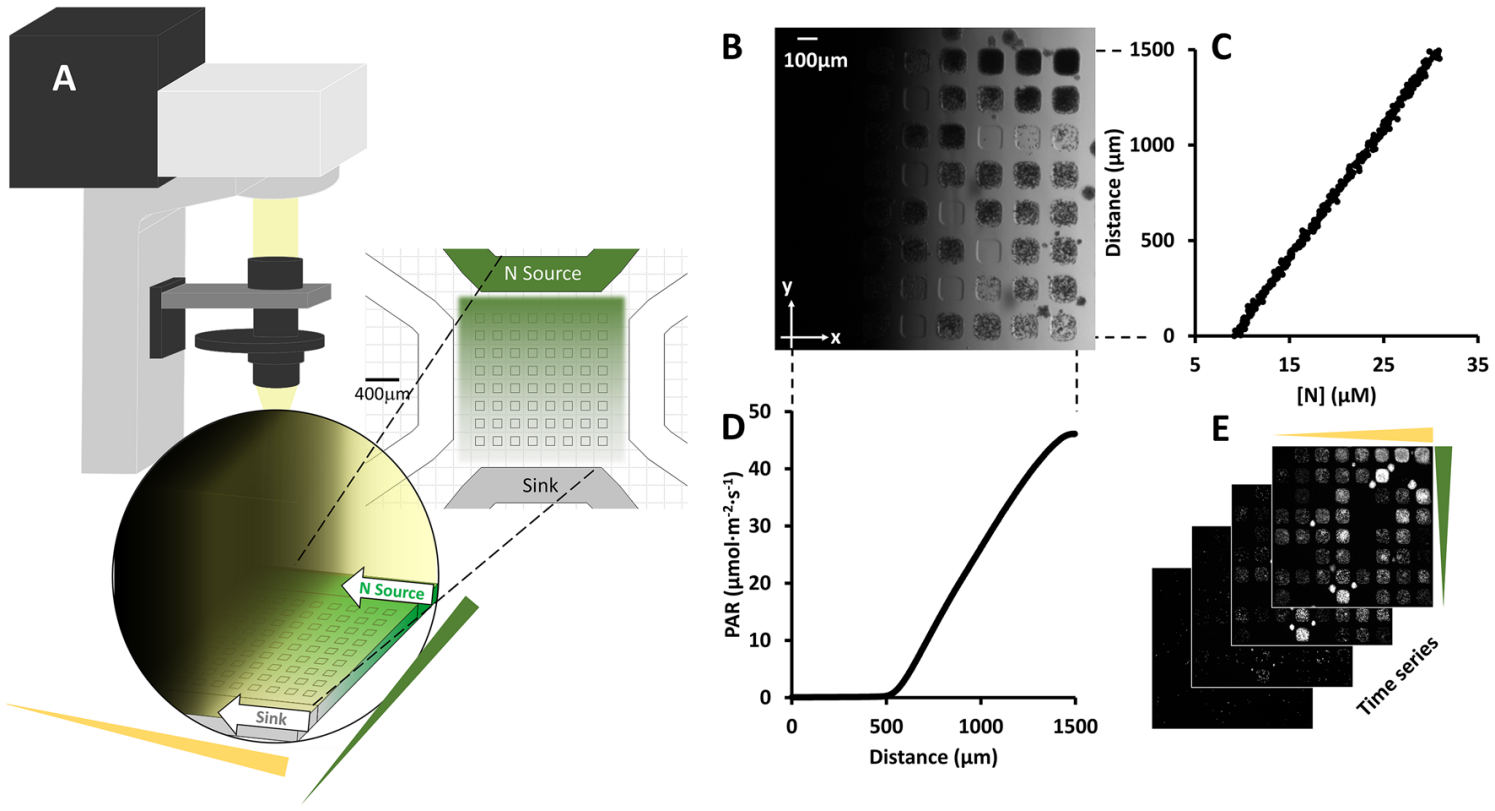
Generation of a dual light and nitrogen gradient for algal cells. (**A**) Schematics of the experimental setup. The transmitted illumination light path of an inverted microscope was modified to provide a light gradient. A hydrogel-based array microhabitat platform was used to provide a nitrogen concentration gradient for algal cells. Here, media with a constant nitrogen concentration and buffer flow through the source and sink channels, respectively, and a nitrogen concentration gradient was established in the array microhabitat via diffusion. The blank side channels were filled with blank media and plugged. (**B**) A bright field image of the 8 × 8 microhabitat array on day 7. The light intensity gradient was in the x-direction and the nitrogen concentration gradient was in the y-direction. *Chlamydomonas reinhardtii* cells were seeded in the array microhabitats on day 0. (**C**) Characterization of the nitrogen gradient in the microhabitat array. Experimental nitrogen concentration ([N]) gradient along the y direction across the microhabitat array. The linear gradient was computed using images taken when the fluorescent dye and blank buffer flow through the source and sink channels respectively. Mean [N] at the horizontal centerline of each row of microhabitats was used as the nitrogen concentration for the row. (**D**) Light intensity gradient in the x-direction as measured by grayscale values of bright field images of the microhabitat array. The PAR (µmol·m^–2^·s^–1^) values were converted from grayscale values using a linear relationship obtained in a previous work^51^. Mean light intensity at the vertical centerline of each column of microhabitats was used as the light intensity for the column. (**E**) Time lapse fluorescence images of the microhabitats under dual light and nitrogen gradients. Images at 0, 14, 28, 42 h are shown here.

A hydrogel-based array microhabitat device in conjunction with a light gradient was used to study algal growth under well-defined physical and chemical conditions (See Fig. 1A). To create a nitrogen gradient, we used a previously developed hydrogel-based chemical gradient generator^48^. We note that ammonium was used as the nitrogen source in this work. The device consists of an 8 × 8 array of microhabitats (each with a size of 100 μm × 100 μm × 100 μm) flanked by two side channels (400 μm W × 200 μm H), patterned on a 1 inch × 3 inch size and 1 mm thick agarose gel membrane (Fig. 1A and Fig. S1). Nitrogen-starved cells were seeded in the microhabitats. The nitrogen gradient was established by flowing medium with known nitrogen concentrations and blank buffer through the top and bottom side channels respectively, creating a nutrient gradient at the location of the array microhabitats via molecular diffusion. The time to reach a steady state nutrient gradient was measured to be about 90 min^48^. The light intensity gradient was generated by modifying the bright field illumination light path of a commercial microscope (Fig. 1A and Ref.^51^). Briefly, the light intensity gradient at the location of the array microhabitat was created by placing a half-moon shaped mask directly below the field iris of an Olympus IX81 microscope.

Figure 1B shows an image of cells growing in microhabitats under a double light and nitrogen gradient, which was characterized in the following way: The nitrogen concentration gradient in the dual gradient experiment is shown in Fig. 1C. To characterize the chemical gradient in the array microhabitat, we flowed a solution of a known concentration of a fluorescent dye in the source channel and blank buffer in the sink channel as described previously^48^. The steady linear gradient computed from the fluorescence image is shown in Fig. 1C. Here, we assumed the nitrogen concentration in the sink and source channels was 5.3 and 35.3 µM respectively, and the concentration of the fluorescent solution was linearly related to the nitrogen concentration. Nitrogen concentrations at the middle line of each row of microhabitats were assumed as the concentration of that row. The light intensity was characterized using a light meter and readouts from a CCD camera. Figure 1D shows an almost linear light intensity profile across the array microhabitat. The maximum value of photosynthetically active radiation (PAR) was approximately 45 µmol·m^–2^·s^–1^ and the minimum was 0.1 µmol·m^–2^·s^–1^ (Fig. 1D). PAR values were converted from the grayscale values measured from brightfield images of the light intensity gradient captured with a CCD camera as described previously^51^. The light intensity of each column was calculated as the average across the width of the microhabitats in that column, as cells were observed to swim freely within single habitats. The platform could provide 64 distinct combinations of nitrogen concentrations and light intensities, which is high throughput as compared to conventional large volume growth systems.

To monitor cell growth dynamics, time lapse fluorescence images were taken every four hours during the 7-day experimental period (Fig. 1E). Cell numbers were measured using the fluorescence intensities in each microhabitat, and were used to calculate the algal cell growth rates.

### Algal growth response to light was enhanced at high nitrogen concentration

**Figure 2:**
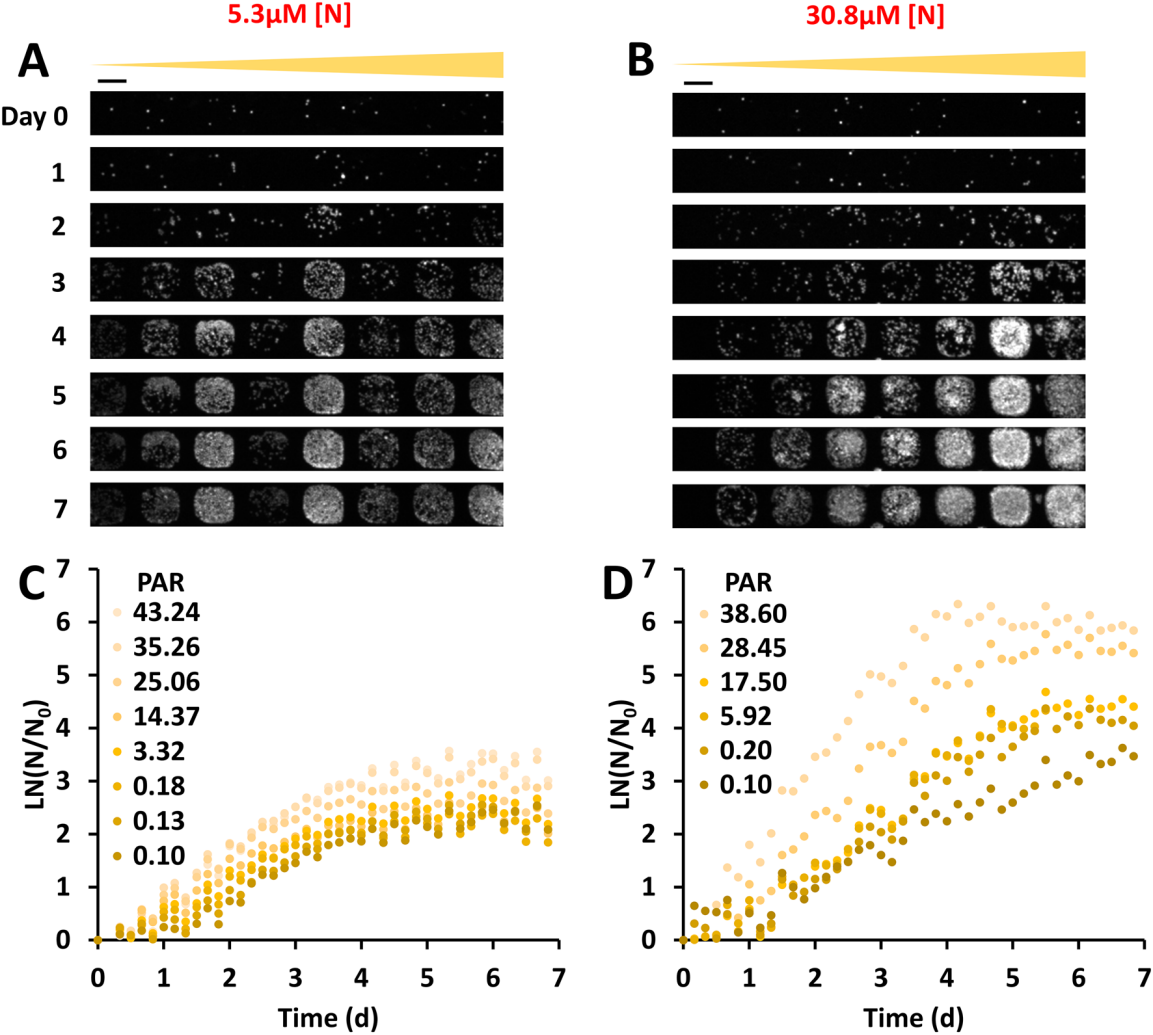
Nitrogen enhanced the algal growth response sensitivity to light. (**A**,**B**) Time sequence of fluorescence images of one row of algal cells under a light intensity gradient with low (5.3 µM, (**A**)) and high (30.8 µM, (**B**)) nitrogen concentration. Contrast was adjusted to show details: Maximum grayscale value was taken as 15,000 for day 0–4 images, and 35,000 for day 5–7 images. The scale bar represents 100 µm. (**C**,**D**) Growth curves of algal cells in the microhabitats at various levels of light intensity for low (**C**) and high (**D**) nitrogen concentrations respectively. Growth was calculated by taking the natural log of cell number N divided by the initial cell number N_0_, as measured by the fluorescence intensity. We note that initial cell seeding was a random process that could result in variations in cell density among microhabitats. This heterogeneity was reduced by normalizing N by the initial cell number, N_0_ for each habitat. Colors of the dots represent light intensities. Photosynthetically Active Radiation (PAR) is expressed in units of µmol·m^–2^·s^–1^. Results shown are from one of the three replicates.

*C. reinhardtii* cells grew very differently under the same light intensity gradient at low versus high nitrogen concentrations (Fig. 2A,B). Cells were starved in medium with 5.3 µM nitrogen before loading them into the microhabitats (“Materials and methods”). When the cells were provided with a low nitrogen concentration of 5.3 µM in the device, no clear response to light was observed (Fig. 2A). In contrast, when 30.8 µM of nitrogen was provided uniformly across all the habitats, cells showed a clear increase in growth rate as light intensities increased (Fig. 2B). Figure 2C,D shows the corresponding growth curves, normalized by the initial cell number that was usually 1–6 cells per habitat due to the random seeding process. Microhabitats without cells were excluded from the analysis. The normalization with respect to the initial cell number revealed a slight growth response to light intensity at 5.3 µM nitrogen (Fig. 2C), which was not apparent from the fluorescence images (Fig. 2A). In the presence of 30.8 µM nitrogen, the growth curves at different light intensities were clearly spread out (Fig. 2D), indicating that cell growth had higher sensitivity to light at the higher nitrogen concentration. Together, the results clearly showed that nitrogen concentration affected algal growth sensitivity to light.

### Algal growth response to nitrogen was enhanced under high light intensity

**Figure 3:**
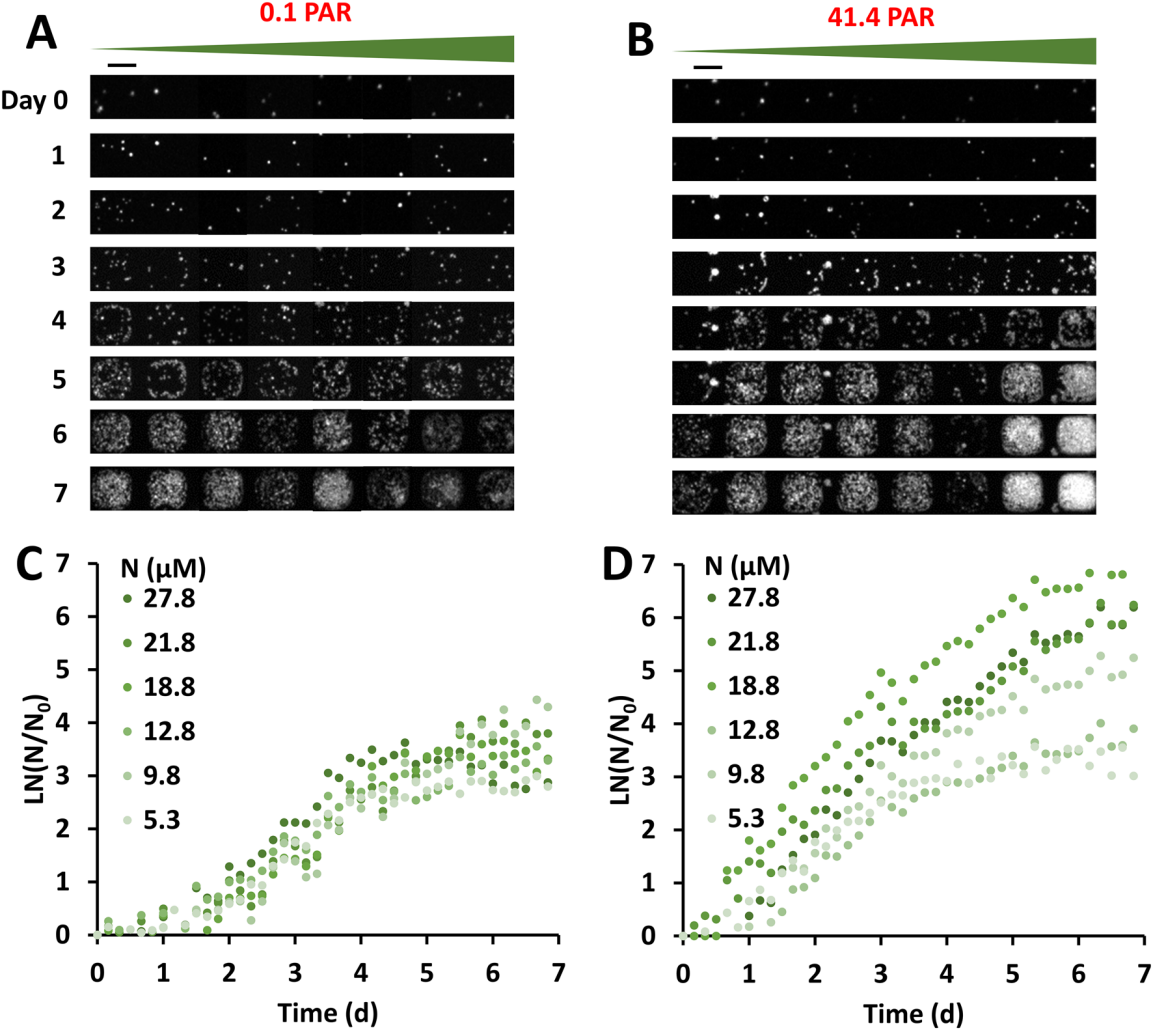
Light enhanced the algal growth response sensitivity to nitrogen. (**A**,**B**) Time sequence of fluorescence images of algal cells in one column of microhabitats (rotated 90 degrees into a row) under a nitrogen concentration gradient with low (0.1 PAR, (**A**)) and high (41.1 PAR, (**B**)) light intensities. Contrast was adjusted to show details: Maximum grayscale value was taken as 15,000 for day 0–4 images, and 35,000 for day 5–7 images. The scale bar represents 100 µm. (**C**,**D**) Growth curves of algal cells at various levels of nitrogen concentration in the microhabitats for low (**C**) and high (**D**) light intensities. Growth was calculated by taking the natural log of cell number N divided by the initial cell number N_0_, as measured by fluorescence intensity. Colors of the dots represent nitrogen concentration. Photosynthetically active radiation (PAR) is expressed in units of µmol·m^–2^·s^–1^. Results shown are from one of the three replicates.

Likewise, *C. reinhardtii* cells grew very differently under the same nitrogen gradient with low versus high light intensity (Fig. 3). Here we did a similar set of experiments as shown in Fig. 2 except under different environmental conditions. We grew cells under a nitrogen gradient (from 5.3 to 27.8 μM) under low (0.1 PAR) versus high (41.4 PAR) intensity. Figure 3A is a time evolution of one row of microhabitats subjected to a nitrogen gradient under low uniform lighting. We see a slight increase in cell growth from low to high nitrogen, but the difference is subtle. This shows that cells have low growth sensitivity to nitrogen (Fig. 3A) under low lighting. Figure 3B is a time evolution of one row of microhabitats subjected to the nitrogen gradient under high light intensity. It is clear from the images that the cells grew faster under high nitrogen concentration in contrast to those under low nitrogen concentration. This trend was more clearly demonstrated when we calculated and plotted the growth rate at various nitrogen concentrations using the images such as those shown in Fig. 3A,B. Figure 3C shows that the growth curves did not spread out when changing the nitrogen concentration from low to high at low light intensity; while Fig. 3D shows that the cell growth curves spread out significantly across different nitrogen concentrations under high light intensity. Together, results in Fig. 3 clearly demonstrated that light intensity enhanced algal growth response to nitrogen.

### Light intensity and nitrogen concentration synergistically influence the growth rate of algal cells

**Figure 4:**
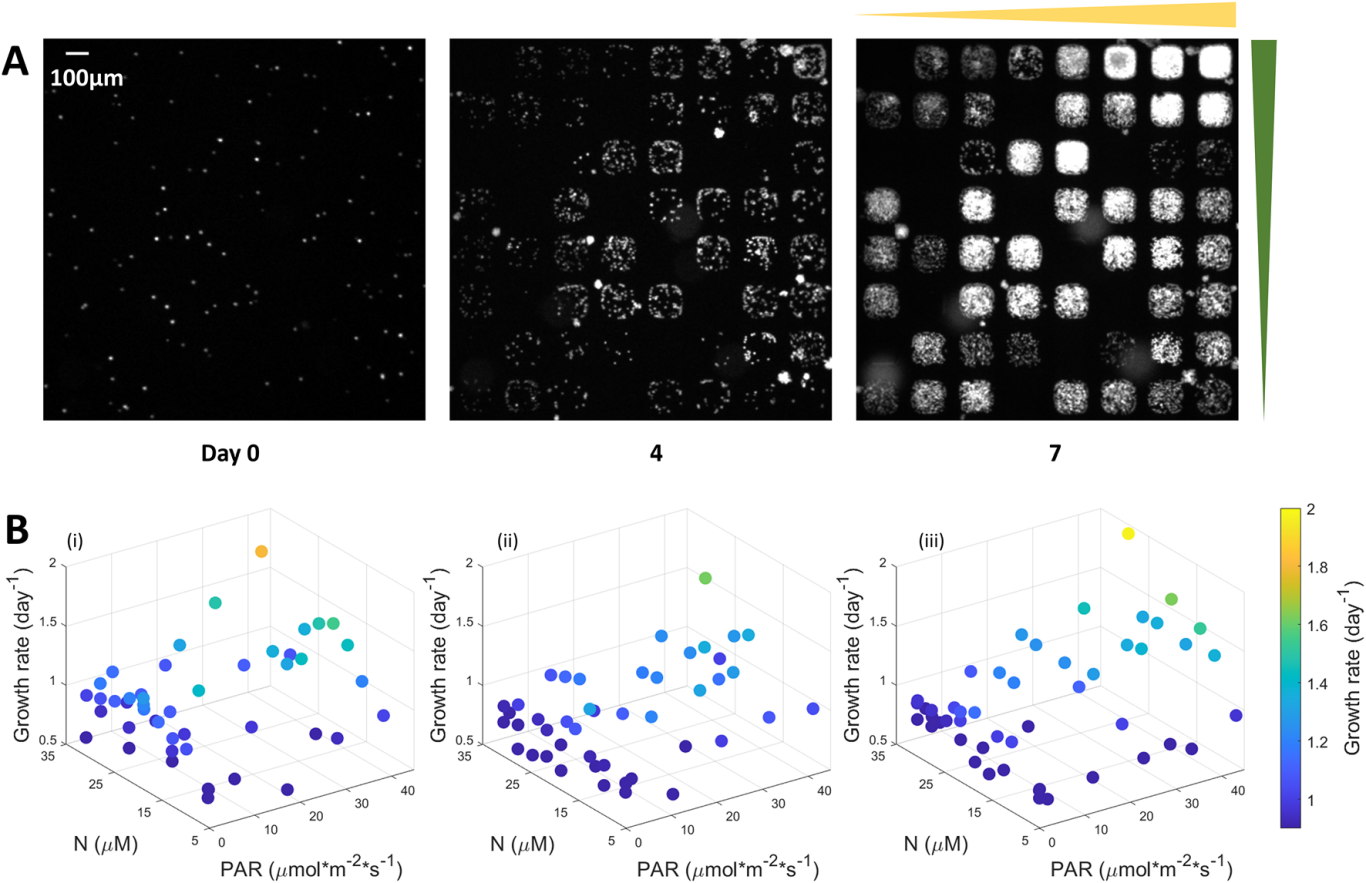
Light and nitrogen synergistically enhanced algal growth. (**A**) Fluorescence images of algal cells growing under dual light and nitrogen gradients (as described in Fig. 1) on day 0, 4 and 7. The nitrogen gradient was 1.3 µM per 100 µm and the light intensity gradient was 2 µmol·m^–2^·s^–1^ per 100 µm. Contrast was adjusted for illustration purposes: the intensity (0–255) corresponds to (0–10,000) of the grayscale of original images for day 0 and 4, and (0–20,000) for day 7. (**B**) 3D plots of cell growth rates in single microhabitats. Results shown in (i–iii) are three sets of replicated experiments. The color of dots represents growth rate as shown in the color bar.

Light and nitrogen were found to synergistically promote algal growth when the algal cells were grown in the array microhabitats in the presence of both light and nitrogen gradients. Here, the nitrogen gradient was generated by flowing medium with 35.3 µM nitrogen in the source channel and medium with 5.3 µM nitrogen in the sink channel. The light intensity was provided by the brightfield light source of the microscope, ranging from 0 to 45 µmol·m^–2^·s^–1^. The synergistic effect can be seen clearly in Fig. 4A, which shows fluorescence images of cells in the array microhabitats. Therein, the upper right corner represents the habitat with the highest light intensity and highest nitrogen concentration. To understand quantitatively how cell growth depends on light intensity and nitrogen concentration gradients, we calculated growth rates of each microhabitat and displayed them in Fig. 4B. Results from three replicated experiments were shown in Fig. 4B. In all three replicates, the data was consistent, and shows that growth rate was highest at high nitrogen concentration and light intensity.

We noticed an interesting parallel between the cell growth response to dual light and nitrogen gradients (shown in Fig. 4) and our previous work where cell growth response was studied under dual nitrogen and phosphorous gradients^48^. Under a single nutrient or light gradient, we used a microfluidic platform to reveal that algal cell growth followed a Monod growth kinetic model^39,51^. Under a dual nitrogen and phosphorous gradient, a multiplicative model fitted to the growth data showed the colimitation by two nutrients (Supplementary Information). The experimental data presented here inspired us to ask whether there is a general growth kinetic model describing algal cell growth under complex physical and nutrient environments.

### Colimitation of algal growth by light and nitrogen: theoretical modeling

We propose a general colimitation growth model for algal cells subjected to both physical and chemical parameters. Previous work in our labs showed that algal cell growth followed Monod growth kinetics when grown with either a nitrogen or a light intensity gradient alone^39,51^. Here, we hypothesized that algal growth response to light or nitrogen was described by Monod kinetics individually, and the synergistic effect of light and nitrogen on algal growth was reflected in a multiplicative term of the model:

1 $$\mu ={\mu }_{max}\left(\frac{L{+L}_{0}}{{K}_{L}+L{+L}_{0}}\right)\left(\frac{\left[N\right]+{N}_{0}}{{K}_{N}+[N]+{N}_{0}}\right).$$

Here, ${\mu }_{max}$ is the maximum growth rate, ${K}_{L}$ is the half-saturation constant of light intensity, and ${K}_{N}$ is the half-saturation constant of nitrogen concentration. We note that the response term to nitrogen is a Monod kinetic model, where ${N}_{0}$ represents stored nitrogen. The response term to light is also a Monod kinetic model, with a storage term ${L}_{0}$. This term is required since we observed residual growth in the absence of light, which comes from the acetate in our media. We note that the concentration of acetate is typically very low in natural body of water, however, it is customary to add it to media in the lab for fast growth. When no acetate was added in media, no residual growth was observed in the absence of light (Fig. S4). Detailed discussion on the effect of acetate on algal growth can be found in the Supplementary Information.

**Figure 5:**
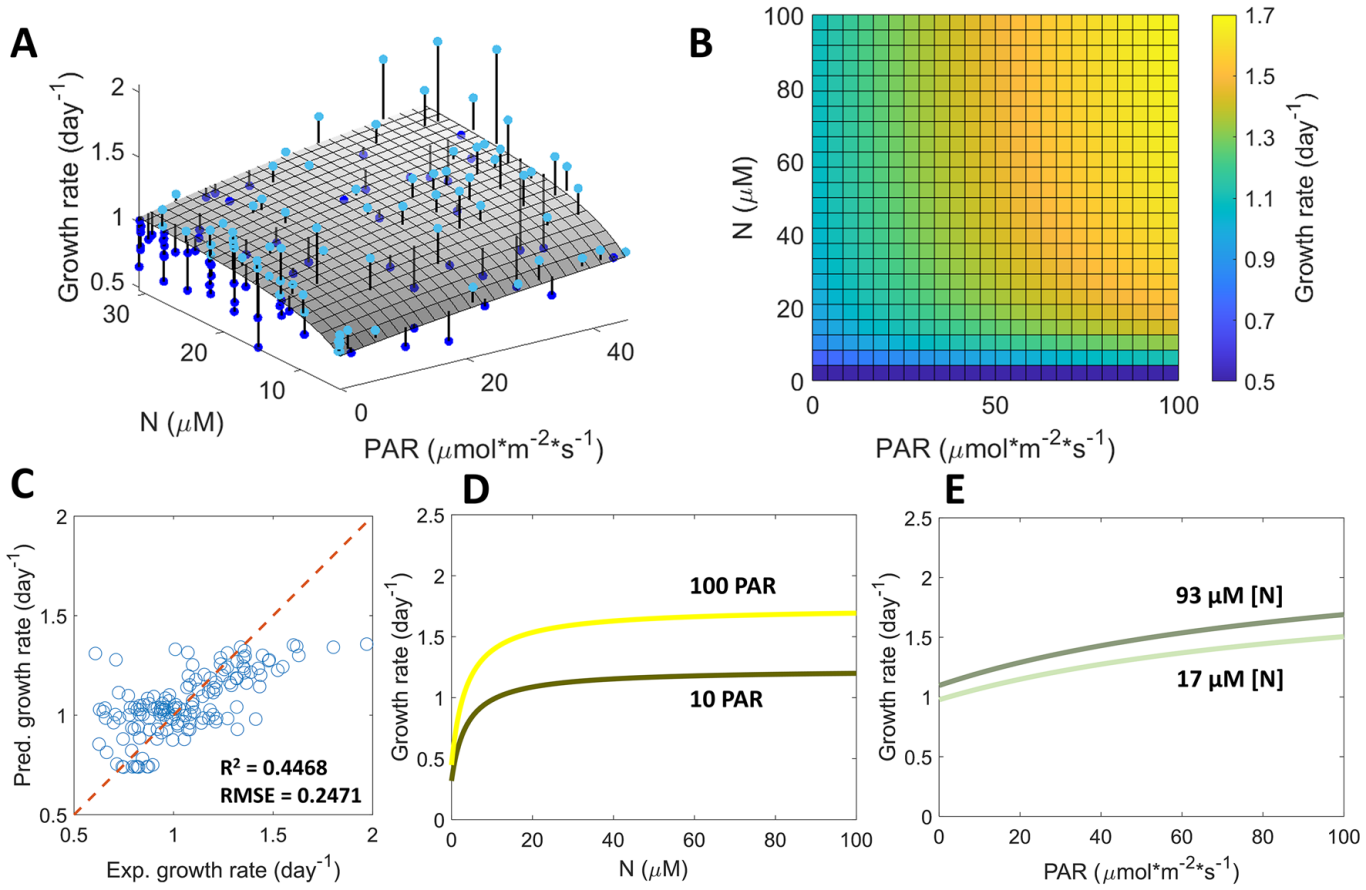
Light and nitrogen co-limit algal cell growth, revealed by a general multiplicative growth model. (**A**) 3D plot of the growth rate as a function of nitrogen concentration and light intensity. The dots are experimental data from three replicates, and the surface is a fit to the proposed general multiplicative Monod kinetics model. Experimental dots are connected to the fitted surface by vertical lines for visualization purpose. Dots above or on the surface are plotted in light blue, while those below are in dark blue. The fitted parameters were L_0_ = 50.8 µmol·m^–2^·s^–1^, K_L_ = 57.2 µmol·m^–2^·s^–1^, and K_N_ = 2.8 µM. (**B**) A lookup map showing the predicted growth rate under wide ranges of light intensities and nitrogen concentrations using the multiplicative model. (**C**) Scatter plots of predicted growth rates from the multiplicative model versus experimental growth rates. (**D**) Predicted algal growth rate as a function of nitrogen concentration under low (10 PAR) and high (100 PAR) light intensities. (**E**) Predicted growth rate as a function of light intensity under low (17 µM) and high (93 µM) nitrogen concentrations.

We fitted Eq. (1) to experimental data under the dual light and nitrogen gradients. During fitting, ${K}_{L}$ and ${K}_{N}$ were kept as free fitting parameters. ${\mu }_{max}$ was fixed to be 2.4 day^–1^, which was the maximum growth rate obtained in the microhabitats previously^39^. ${N}_{0}$ was set to 0 µM, as the cells were starved in low N concentration media prior to experiments (See Supplementary Information). In the case of acetate, ${L}_{0}$ was left as a free parameter. In the case without acetate, ${L}_{0}$ was set to 0 µmol·m^–2^·s^–1^ because (1) we observed no growth in the absence of both light and acetate and (2) fitting Eq. (1) to growth data obtained in the absence of acetate gave a best-fit value of ${L}_{0}$ close to 0 µmol·m^–2^·s^–1^ (p-value = 0.18, Supplementary Information). The fitted surface to Eq. (1) is plotted together with experimental data in Fig. 5A. The best fit parameters were ${L}_{0}$ = 50.8 µmol·m^–2^·s^–1^, ${K}_{L}$ = 57.2 µmol·m^–2^·s^–1^, and ${K}_{N}$ = 2.8 µM, with standard deviations of 7.9 µmol·m^–2^·s^–1^, 8.8 µmol·m^–2^·s^–1^, and 0.4 µM, respectively. Distributions and correlations of the fitted parameters are shown in Fig. S3.

Colimitation of algal growth by light and nitrogen was shown by the microhabitat dual gradient experiments and subsequent fitting to a general multiplicative growth model. While growth response to a single resource has often been described via Monod kinetics, here, we showed that growth response to a physical and a chemical parameter could be described by the multiplication of the two Monod kinetics terms with respect to each single resource. Estimated half saturation constants of light and nitrogen in previous studies were 81.4–215.5 µmol·m^–2^·s^–1^ and 2.2–17 mM respectively^29,37^. These values were larger as compared to the half-saturation constants obtained from our microhabitat platform and varied in a wide range. The differences could come from the self-shading effect, light spectra and varying nutrient concentrations in large scale experiments. We also note that the half-saturation constants here depended on the history of the cell (starved or not), the provided light spectrum, and the form of nitrogen. Previous studies using non-starved cells gave ${K}_{L}$ and ${K}_{N}$ based on single light and single nitrogen gradients in the microhabitat platform to be 1.9 µmol·m^–2^·s^–1^ and 1.2 µM, respectively^39,48^, which is smaller than those found in this work. In addition, we also compared different forms of models, including a multiplicative form with only ${\mu }_{0}$, ${K}_{L}$, and ${K}_{N}$ as fitted parameters and a law of minimum form of the growth kinetic model (see Supplemental Information). It was seen that the proposed model in Eq. (1) had better goodness of fit as compared to the other forms.

### Predicting growth rate under various light intensities and N concentrations using the colimitation model

The general colimiting model can be used to predict and understand how light and nutrient synergistically control the growth of algal cells. Using the fitted model, a look up map was generated that predicts algal growth rate given the light intensity and the nitrogen concentration (Fig. 5B). In general, growth is suppressed when either N is under 20 uM, or light intensity is under 20 PAR. This look up map could be used to make predictions on algal growth trends under various light and nitrogen conditions (Fig. 5C). For example, one could predict the effect of environmental drivers on algal growth in freshwater bodies, where light intensity and nitrogen conditions could vary seasonally, as well as geographically. The Finger Lakes in upstate New York, USA serves as an example. In the 2020 sampling season, the light intensity at 6 m depth and nitrogen concentration at the lake surface in Cayuga Lake (South Shelf Site) were 0.1 PAR and 93 µM respectively, while the values for Hemlock Lake (Mid Site) were 100 PAR and 17 µM (data was taken from the Citizens Statewide Lake Assessment Program reports, conversion from clarity measurements to light intensities could be found in SI). Comparing the growth response to nitrogen concentration at the two different light intensity levels in Fig. 5D, it was shown that nitrogen concentration would be a more important driver for algal growth in Hemlock Lake (100 PAR) than in Cayuga Lake (0.1 PAR). In addition, comparing the growth response to light intensity at the two different nitrogen concentration levels in Fig. 5E, it was seen that light intensity would be a slightly more important driver in Cayuga Lake (93 µM) than in Hemlock Lake (17 µM). We note that in natural lakes, the spectrum of light and its attenuation down the water column, the forms of nitrogen comprising Total Nitrogen (TN), and the blooming species are not exactly accounted for in our experiments supporting the model. However, this example shows the potential use of the experimental method and the model to make relevant predictions to some level of generalization. We emphasize here that our multiplicative model provides a basic understanding of how environmental cues impact algal growth in a simplified setting, focusing on the physiology of algae rather than on ecological interactions. It does not include many complexities in natural setting such as microbial communities, wind, and turbulence. In natural aquatic microbial communities, there are heterotrophic bacteria associated with the algae that can make use of the organic carbon from algal exudates^52^. These bacteria could increase the availability of macronutrients (e.g. nitrogen and phosphorous)^53–55^ and micronutrients (e.g. iron and vitamin B_12_)^56–59^ for the algae. Therefore, when considering the existence of these bacteria in predicting algal growth under varying nutrient conditions, we expect the kinetic constant to be lower for the nutrients that are made more readily available or cycled within the microenvironment by the bacteria. Furthermore, to model the dynamics of the microbial community, various approaches could be applied depending on the available knowledge of the system^52^. For example, consumer-resource models, inspired by MacArthur’s consumer-resource model, can be used to predict the dynamics of algae, bacteria, and resources, with the availability of information on resource uptake rates.

## Discussion

Mathematical modeling of algal growth under the influence of various physical and chemical factors in their microenvironment is important for understanding the ecology and evolution of phytoplankton-related aquatic microbial communities, controlling harmful algal blooms, and improving biofuel production. Using a microfluidic platform, we found that algal growth response to environmental parameters was described by a multiplicative Monod kinetic model, consisting of the Monod growth kinetics due to light multiplied by the Monod growth kinetics due to nitrogen. Interestingly, we found that the contributing term to the multiplicative model for both a physical and a chemical parameter was similar, given that both terms can be described by Monod kinetics. The proposed model from our experiment reflects the intrinsic growth response of algal cells under the precisely applied environmental conditions experienced by all cells in the population, thus, we expect the model to be independent of the experimental system. To take a step further, we hypothesize a general multiplicative model by including all potential independent, non-substitutable physical and chemical resources:

2 $$\mu ={\mu }_{max}{\prod }_{i=1}^{n}\left(\frac{{E}_{i}+{{E}_{i}}_{o}}{{{K}_{E}}_{i}+{E}_{i}+{{E}_{i}}_{o}}\right).$$

Here, ${E}_{i}$ stands for the i-th factor of the $n$ number of independent colimiting factors such as light, nitrogen, phosphorous, and CO_2_^28,60, 61^. ${{K}_{E}}_{i}$ is the half saturation constant for the i-th environmental factor, and ${{E}_{i}}_{o}$ is the storage term. ${\mu }_{max}$ is the maximum growth rate at saturation. Future experiments will be needed to develop a true general growth model in a complex environment. “Independent” and “non-substitutable” resources are those that are biochemically mutually exclusive in general, and was proposed by Saito et al. as “Type I independent nutrient colimitation”, as compared to “Type II biochemical substitution collimation” and “Type III biochemically dependent colimitation”^28^. An example of substitutable elements is zinc and cobalt, which could substitute for an active site in the carbonic anhydrases in marine diatoms^62,63^. Also in this system, zinc and carbon are considered dependent substrates, as the half-saturation constant for bicarbonate increases when zinc becomes limiting^26,62^. Although all intracellular processes are connected to some extent, here, “independent” and “non-substitutable” are used to define resources without clear and direct biochemical interactions, such as nitrogen and phosphorus^60^, nitrogen and light^60^, nitrogen and carbon^61,64^, iron and cobalt^65^, iron and zinc^66^, iron and phosphorous^67^, and iron and vitamin B12^68^. While our proposed model addresses the independent and non-substitutable factors, it is likely that different type of colimitation and their combinations occur in natural waters.

In this work, we demonstrated an array microhabitat device for algal cell studies under well-defined microenvironments and the results are ideal for data driven theoretical modeling. The key innovations of our microfluidic platform include allowing for growing free swimming algal cells within individual microhabitats, and the growth dynamics under controlled multiple resources can be continuously monitored in real time instead of end point measurements. In addition, the self-shading effect is minimized in the nanoliter-sized microhabitats. This platform can be easily adapted to studies of photosynthetic microbes under two nutrient gradients (e.g. nitrogen and phosphorous) alone or with a light gradient. We note that the generated light and ammonium gradients were kept at low levels for the investigation of colimitation effects of the two factors. The range can be easily extended by varying the lamp power output and the concentration of nitrogen in the medium perfused from the side channels. By using specially designed LED light, we can also provide light spectrum that mimics natural light. Our platform is also amenable to studies of other cellular behavior such as competition in microbiomes or cell motility. While this small-scale microfluidic device provided a way to precisely define a complex microenvironment for cells, the downside is that there was a small number of cells in each habitat. Although this increased the heterogeneity of growth dynamics parameters in neighboring habitats and technical replicates, it opens up the opportunity to study how cell-to-cell phenotypic heterogeneity affects growth dynamics under nutrient and light gradients in future work, which is often not possible at large population level^69^. The next level of inquiry will be whether growth rate variations of algal (cyanobacterial) cells provide an advantage for their successful survival in the natural environment, as well as their response to potential treatment of HABs.

Using the microhabitat platform described here, we observed synergistic effects of light and nitrogen on algal growth, where the increase in one resource resulted in the enhanced growth sensitivity to the other. Light is an important energy source for photosynthetic microbes like microalgae. Energy harnessed from light can be used for the biosynthesis of structural and functional components of the cell, contributing to cell proliferation. Meanwhile, nitrogen is one of the most important nutrients for algae, contributing to the synthesis of amino acids, proteins, nucleic acids, and chlorophyll. The interplay between light and nitrogen has been studied from two major perspectives: (1) how nitrogen starvation affects photosynthesis, and (2) how increase in the intensity and presence of light affects nitrogen metabolism. Upon the removal of nitrogen, proteins and pigments related to photosynthesis decrease in abundance, including RuBisCO, a key enzyme in the Calvin cycle, and chlorophyll, the pigment for capturing photons, which leads to reduced efficiency of light utilization^70–73^. Our results in the microhabitats indeed showed that cell growth was less sensitive to light at low nitrogen concentrations, indicating a suppressed photosynthetic capacity. Increased sensitivity to light in the presence of nitrogen as compared to no nitrogen has been shown either by -omics response or by photosynthetic functional measurements^70,71^. Here, we showed that growth rate, as a result of all cellular processes combined, was more sensitive to light under the high nitrogen concentration. In addition, increase in light intensity has been found to induce responses in various metabolic processes including photosynthesis, as well as amino acid, fatty acid, and nucleotide biosynthesis, where nitrogen metabolism in specific is also known to be affected by light^74–76^. Various forms of nitrogen can be utilized by algal cells including nitrate, nitrite, ammonium, and some organic forms, among which ammonium is often the preferred form. Ammonium is assimilated through the glutamine synthetase-glutamate synthase pathway, and the products of which can be used for the biosynthesis of macromolecules for cell function and proliferation. The presence of light was found to contribute to higher activity of glutamate synthases^77^, thus leading to more efficient nitrogen utilization for growth. For other inorganic nitrogen sources such as nitrate and nitrite, they need to be reduced stepwise through nitrate reductase and nitrite reductase to ammonium before being further assimilated by the cells^78,79^. This indicates that it is likely that the kinetic constant will be lower if we use nitrate or nitrite as the single nitrogen source, due to proteome allocation requirements to the nitrate/nitrite assimilation pathway and the additional biochemical steps involved in the process. In addition, it was found that ammonium inhibits the activity of nitrate reductase and high affinity nitrate/nitrite transporters^79–81^. Therefore, if mixed inorganic nitrogen sources are present in the environment, algae likely assimilate ammonium first before nitrate and nitrite. Nitrate, nitrite, and ammonium are examples of substitutable resources. Future experimental and modeling work will be needed to model the growth transitions between these substitutable nitrogen sources and energy sources (e.g. light). The experimental observations presented in this paper begin to reveal the interactions between nitrogen metabolism and photosynthesis machineries. A systematic study will be needed to reveal the molecular mechanisms underlying the interplay between photosynthesis and metabolism of nitrogen in algal response to complex light and nitrogen conditions.

In response to nitrogen stress, algae such as *C. reinhardtii* modify their metabolism to accumulate high amounts of storage molecules including triacylglycerol (TAG), along with the reduction in photosynthesis. This makes them a promising candidate for biofuel production^7,9, 70, 82–85^. While nitrogen deprivation studies have mainly compared milli molar ammonium to the nitrogen-deprived state, here, we found that an increase of nitrogen concentration in the micro molar range could clearly affect cells utilization of light for growth. This information could possibly be used to search for a nitrogen condition with high TAG accumulation as well as a reasonable growth rate to optimize biofuel production. In addition, optimizing biofuel production requires further investigation of the effect of acetate, where experiments could be performed to figure out ${L}_{0}$ proposed in Eq. (1) as a function of acetate concentration.

## Materials and methods

### Cell culture and preparation

Wild type *C. reinhardtii* strain CC-125 was obtained from the Stern Laboratory at the Boyce Thompson Institute of Plant Research on the Ithaca campus of Cornell University. Cells were maintained in 10% TAP (tris acetate phosphate) medium, which has one tenth of the acetate concentration as compared to the standard TAP medium. The 10% TAP medium was composed of 2 mM Tris, 1.7 mM Acetate, 0.68 mM K_2_HPO_4_, 0.45 mM KH_2_PO_4_, 7.5 mM NH_4_Cl, and other salts including 0.34 mM CaCl_2_), and prepared using an established protocol^86^ with trace metal elements concentrations as described in Hunter et al.^87^ A 5 mL cell culture was maintained in 15 mL glass tubes in a temperature-controlled incubator at 25 °C without shaking (New Brunswick Innova 44, Eppendorf) under a continuous illumination of 20 μmol·m^–2^·s^–1^ using LED light (4000 K, Commercial Electric). For experiments in Tris-Minimal medium (TM, TAP without acetate), cells were maintained in a similar manner with TM instead of 10% TAP.

To make medium with low N concentrations, the NH_4_Cl in the Beijerinck’s solution in the 10% TAP or TM recipe was replaced by NaCl. 750 mM NH_4_Cl stock solution was made and added to the medium to achieve final N concentrations in the micro molar range. The medium with no NH_4_Cl stock solution added was referred to as 10% TAP-N or TM-N, which had the lowest nitrogen concentrations of 5.3 uM due to the ammonium salt in the trace element solution in the recipe. NH_4_^+^ was the only nitrogen source in the medium, thus its concentration was taken as the nitrogen concentration.

To set up experiment in the microfluidic platform, cells were prepared via three steps: (i) a 20-day old maintenance culture was diluted 10X into 5 mL of fresh 10%TAP medium; (ii) the newly transferred culture was kept for 5 days before being centrifuged and washed twice, and then diluted 5X into 5 mL of 10%TAP-N medium; (iii) two days after limiting cells of nitrogen, the culture was concentrated 10X to a final density of about 10^6^ cells per mL and used to seed the microhabitats. All the glassware for the nitrogen-limitation experiment was washed with 10% HCl to remove possible nutrient residuals. Experiments in TM were conducted following the same procedures with TM and TM-N.

### Microfluidic device design, fabrication, and assembly

The microfluidic device design consists of an array of 8 × 8 microhabitats (100 μm × 100 μm × 100 μm each) surrounded by two sets of side channels (400 μm W  ×  200 μm H) imprinted in agarose gel, and was proved in previous work to provide stable and well-controlled single and dual chemical gradients via molecular diffusion^48^.

A two-layer SU8 negative photoresist photolithography process was used to fabricate the silicon master with the desired pattern. The pattern was transferred to a 1 mm film of agarose gel by pouring 3% dissolved agarose in 1X PBS (Phosphate-Buffered Saline) onto the silicon master and letting it cure under room temperature. The gel was then soaked in 10% TAP-N medium overnight.

To assemble the device, 200 uL cells (1 × 10^6^ cells/mL) were seeded into the agarose gel with patterned microhabitats. The gel was then sandwiched between a glass slide and a Plexi glass manifold and clamped using a metal frame and screws to seal the microhabitats and side channels. After initial seeding, there were usually 1 to 6 cells in each microhabitat. Empty microhabitats due to randomness of the seeding were omitted in the data analysis, as well as microhabitats in which cells that had been trapped in the surrounding gel during seeding started to grow into the microhabitat throughout the experiment.

### Experimental setup and gradient generations

The microfluidic device was kept on the microscope stage throughout the experiment (7 days). One set of the side channels (top and bottom) was used for medium perfusion and nitrogen gradient generation, while the other set had ends plugged to prevent evaporation. A constant flow rate at 0.7 µL/min through the side channels was maintained by a syringe pump (KDS230, KD Scientific, Holliston, MA) and two 10 mL syringes (Exelint International Co., Redondo Beach, CA). For the nitrogen gradient experiment, the syringe connected to the top channel was filled with 10% TAP with 35.3 μM N, while the syringe connected to the bottom channel was filled with 10% TAP-N. For the lowest nitrogen (5.3 µM) condition, the source and sink channels were both perfused with 10% TAP-N, with habitats either under a light intensity gradient, or under dark (0 PAR).

The transmitted light path of an inverted microscope (Olympus IX81, Center Valley, CA) was modified to generate a light gradient as described previously^51^. Briefly, a half-moon mask was inserted into the light path, perpendicular to the light beam from the bright field halogen lamp (Olympus U-LH100L-3), which resulted in a light gradient with a light–dark transition region near the middle of the sample plane. The microscope room temperature was controlled at 25 °C.

### Imaging

Microscopic images were taken by an EMCCD camera (ImagEM X2 EM-CCD camera, Hamamatsu Photonics K.K.). For fluorescence imaging of *C. reinhardtii* cells, a fluorescence lamp (X-Cite 120PC Q, Excelitas Technologies Corp.), a 488/10 nm single bandpass excitation filter (Semrock, Rochester, NY), and a 440/521/607/700 quad-bandpass emission filter (Semrock, Rochester, NY) were used. During the 7-day experiment period, fluorescence images were taken every 4 h with 50 ms exposure using the cellSens imaging software (Olympus Life Science).

### Data analysis

Fluorescence intensities of microhabitats were measured using the software ImageJ and the background was subtracted for use as a measure proportional to cell number, as validated by cell counts in bright field images. The growth rates were obtained by fitting n consecutive data points of ${\text{ln}}\frac{N}{N0}$ versus time in the growth curve of each microhabitat to a linear function, and finding the maximum after 1.3 days (Fig. S2). For our data analysis, n = 9 was chosen because (1) it reduces the noise in measurements, as the calculated growth rates versus time curves flattened out as n increased from 5 (Fig. S2A), and (2) further increasing n until 25 did not overly change the maximum growth rate values obtained, indicating only marginal benefit in noise reducing for n values larger than 9 (Fig. S2B,C).

Growth kinetics model fitting was done using a bootstrapping method on three replicate experimental datasets. For each bootstrapping run, a growth rate matrix at the tested combinations of light and nitrogen conditions was constructed by randomly sampling from the three datasets with a weight given by the reciprocal of the squared standard error from 4 points around each maximum growth rate. The constructed growth rate matrix was then used to fit the multiplicative model (Eq. 1) using nonlinear least square fit function in MATLAB, and the fitting parameters (${L}_{0}$, ${K}_{L}$, and ${K}_{N}$) were recorded. This process was repeated 1000 times, which yielded a distribution for each parameter (Fig. S3). The estimated parameters were calculated by taking the average of the 1000 values.

### Supplementary Information

Supplementary Information.

Supplementary Video 1.

## Data Availability

All data are available in the main text or the supplementary materials.
