# Supplementary material for: A high throughput array microhabitat platform reveals how light and nitrogen colimit the growth of algal cells
Source: Sci Rep. 2024 Apr 29;14:9860. doi: 10.1038/s41598-024-59041-3 (PMC11058252; doi:10.1038/s41598-024-59041-3)
Supplement: Supplementary file 1 — Supplementary Information. [file 41598_2024_59041_MOESM1_ESM.pdf]

Supplementary Materials for

A high throughput array microhabitat platform revealed that light and nitrogen colimited the growth of algal cells

Fangchen Liu *et al.*

*Corresponding authors. Email: mw272@cornell.edu, giometto@cornell.edu

This PDF file includes:

Supplementary Text
Figs. S1 to S5
Movie S1 Caption
Supplementary References

Other Supplementary Materials for this manuscript include the following:

Movie S1

Supplementary Text

A general multiplicative model considering acetate and nutrient storage

Acetate is an organic carbon source that can provide energy to *C. reinhardtii* cells, which likely affect algal growth response to light. We note that the presence of acetate in the natural environment is typically very low. So, in our experiments, we used a lowered acetate concentration as compared to the normal TAP medium. It has been shown that microalgae like *C. reinhardtii* can have different trophic modes depending on the type of carbon and energy sources available [1, 2]. Cells are known to perform mixotrophic growth in presence of both acetate and light, where both inorganic and organic carbon could be used as carbon sources, and both organic carbon and light as energy sources. In algal cells, acetate is incorporated into acetyl coenzyme A (acetyl-CoA), which then goes into the mitochondrial tricarboxylic acid (TCA) cycle for respiration and ATP production (energy source) or into the glyoxylate cycle for gluconeogenesis (C source)[3]. The presence of acetate in the growth medium was found to stimulate growth, promoting respiration while reducing chlorophyll content and photosynthesis capacity [1, 4]. Therefore, as compared to growth in the absence of acetate, cells growing in mixotrophy could have higher growth rate under the same light condition and appear less sensitive to changes in light. In addition, the presence of acetate alone could fulfill the requirement of nitrogen assimilation on both light and CO_2 [3, 5]. Thus, acetate could be viewed as a substitute for light and carbon, which is likely an independent growth limiting factor from nitrogen.

To look at the impact of organic carbon on the algal growth response to light, we performed experiments in the microhabitats using Tris-Minimal (TM) medium under a light gradient, where no acetate was provided, and compared to cells growing in 10%TAP (Fig. S4, 10%TAP-N and TM-N refer to media with no NH4Cl stock solution added, and the nitrogen concentration was 5.3μM in both conditions). It was found that removal of the organic carbon resulted in no baseline growth at PAR=0 (Fig. S4F), indicating photoautotrophic growth where inorganic carbon (CO_2) was the only carbon source, and light the only energy source. Interestingly, cells in TM responded more sensitively to light as compared to those in 10%TAP, as shown in Fig. S4. Therefore, in the growth model, a storage term L_0 was added to address the effect of acetate. In the literature, organic carbon has been mostly treated as an independent factor to light either in a multiplicative manner [6-9], where the kinetic terms for the organic carbon and light were multiplied, or in an additive manner [10, 11], where the contributions from phototrophic and heterotrophic growth were added together. Here, based on the comparison between the two conditions in TM and 10%TAP, we proposed to describe the effect of acetate as L_0 that addressed the decreased growth sensitivity to light, as well as the elevated growth rate as compared to photoautotrophic growth at the same light intensities (Eqn. 1). These growth responses agreed with the growth and metabolic behavior observed in large scale cultures. For the light gradient experiment in TM-N, Eqn. 1 can be written as:

$$\mu = \mu_{max}' \frac{L+L_0}{K_L+L+L_0}. \qquad \text{(S1)}$$

Here, μ_{max}' is the growth rate at saturated light intensity at 5.3μM nitrogen and was fixed to be 0.55day^{-1}. Fitting Eqn. S1 gave L_0 = 1.6±1.3μmol·m^{-2}·s^{-1} and K_L = 15.4±4.3μmol·m^{-2}·s^{-1}. The obtained value of L_0 was not significantly different from 0 (p-value = 0.18). Therefore, when fitting Eqn.1 to the combined datasets obtained with acetate (in 10%TAP) and without (in TM), L_0 was kept as a free positive parameter for the former, while set to 0 for the latter. Note that to figure out L_0 as a function of acetate concentration, experiments on a range of acetate conditions would be required.

In addition to 1) light and nitrogen, a physical factor and a chemical factor independently colimiting algal growth, and 2) acetate and light, a chemical resource affecting the effect of physical light, the effect of phosphorous and nitrogen, two chemical resources, on algal growth has been explored in this array microhabitat platform with dual chemical gradients. It was found that phosphorus and nitrogen synergistically promoted cell growth [12]. The observed non-zero residual growth at [P],[N]=0μM led to the hypothesis that cells had stored phosphorus and nitrogen available despite the starvation on the two nutrients ahead of the experiments. Therefore, the model we proposed to describe the nitrogen and phosphorous colimited growth is:

$$\mu = \mu_{max} \cdot \frac{[P]+P_0}{K_P+[P]+P_0} \cdot \frac{[N]+N_0}{K_N+[N]+N_0}. \quad \text{(S2)}$$

Here, P_0 and N_0 can be understood as equivalent external concentrations representing nutrient storage. Fitting equation S2 with fixed μ_{max} gave P_0 = 8.0±2.1μM, N_0 = 0.97±0.89μM, K_P = 10.2±2.2μM, and K_N = 0.37±0.35μM. The fitting results were shown in Fig. S5 (A-B). An alternative way to address the observed residual growth is to include a μ_0 instead of P_0 and N_0 in the model, as shown in the following equation:

$$\mu = \mu_{max} + \mu_{max} \cdot \frac{[P]}{K_P+[P]} \cdot \frac{[N]}{K_N+[N]}. \quad \text{(S3)}$$

However, model as described by Eqn. S2 provided better goodness of fit, shown by the reduced Akaike information criterion value, as compared to Eqn. S3 (Fig. S5C-D).

Taken together, the growth contribution from acetate and nutrient storage discussed above, assuming that (1) light and nitrogen independently colimit cell growth, (2) acetate only affects cell response to light, and (3) cells keep some level of available nitrogen under starvation, a general multi-resource algal growth kinetics model can be constructed as Eqn. 1, where [P] did not appear because it was provided at concentrations much higher than K_P in nitrogen and light colimitation experiments. The fit of Eqn. S2 gave a value of N_0 compatible with zero (p-value = 0.28), therefore, N_0 = 0μM was used for fitting Eqn. 1.

Some alternative forms of growth models were also looked at including:

$$\mu = \mu_0 + \mu'_{max} \cdot \frac{L}{K_L+L} \cdot \frac{[N]}{K_N+[N]} \quad \text{(S4)}$$

$$\text{and } \mu = \mu_0 + \mu'_{max} \cdot min\left(\frac{L}{K_L+L}, \frac{[N]}{K_N+[N]}\right). \quad \text{(S5)}$$

Here, a distinct μ_0 was included to address baseline growth. Eqn. S4 and S5 were fitted to the data in a similar way to Eqn. 1. Akaike information criterion (AIC) was used to compare the goodness of fit between models with different number of fitting parameters. The calculated AIC for Eqn. S4 and S5 were -293 and -291 respectively, both higher than -436 obtained for Eqn. 1. This showed that the model presented in Eqn. 1 performed better in fitting as compared to other possible forms.

Estimation of lake light intensities from clarity measurements

Light intensities in lakes were estimated from clarity measurements by converting clarity to light extinction coefficient and assuming the surface light intensity. According to the reports from the Citizens Statewide Lake Assessment Program, in the 2020 sampling season, Cayuga Lake (South Shelf Site) and Hemlock Lake (Mid Site) had mean clarity of 2.2meters and 4.4meters respectively. Clarity was measured as Secchi disk depth (Z_{SD}), which could be converted to the extinction coefficient of solar radiation (k) by: k=1.7/ Z_{SD} [13, 14]. Then the light intensity (I) at certain depth (z) in water could be calculated as I(z) = I_0*exp(-k*z), where I_0 is the irradiance just underneath water surface. So, at the same depth of 6m under the lake surface (assuming surface light intensity to be 1000PAR), due to the clarity difference, the light intensity in Cayuga Lake was calculated as roughly 0.1PAR, while that in Hemlock Lake, 100PAR

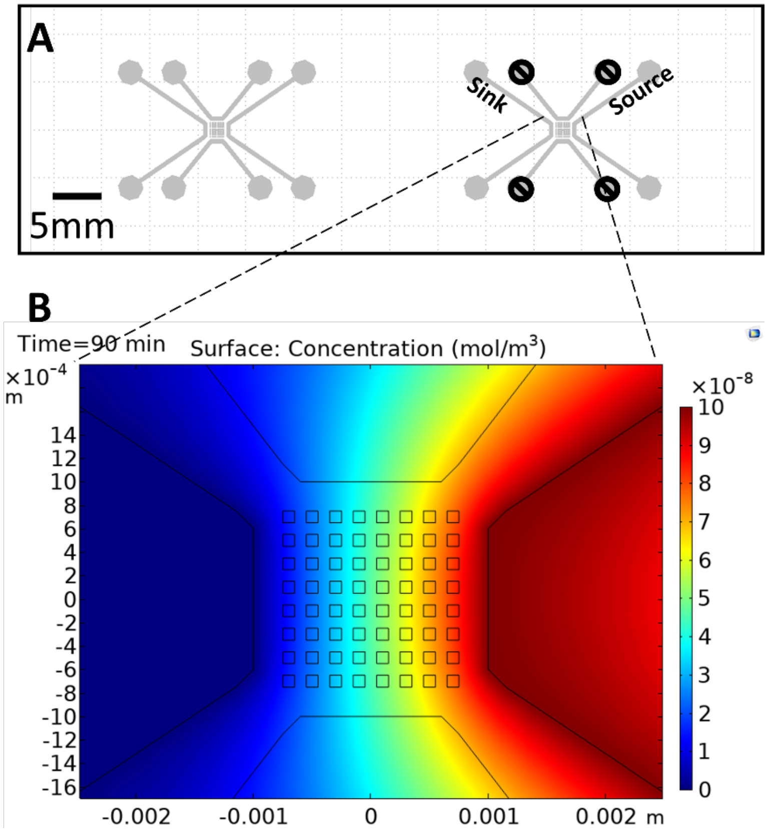

Fig. S1.

Array microhabitat device with chemical gradient generation. (A) Layout of two devices in parallel on a chip. The device on the right shows the generation of a single chemical gradient by perfusing medium with a target chemical in the source channel and blank medium in the sink channel. The other two side channels are filled will blank medium and plugged. Note that this device has the potential to generate dual chemical gradients, but only one gradient is generated and used in our experiments presented in this manuscript. **(B)** Equilibrium chemical concentration field at time t = 90 min from a COMSOL simulation, where the concentration of the diffusive chemical species was fixed as $1*10^7$ mol/m3 and 0 at the right and left channel respectively, and everywhere else was 0 to start with (at t=0). The diffusion coefficient of the fluorescence dye (fitc) in water was used for direct comparison with experimental results (Fig. 1C).

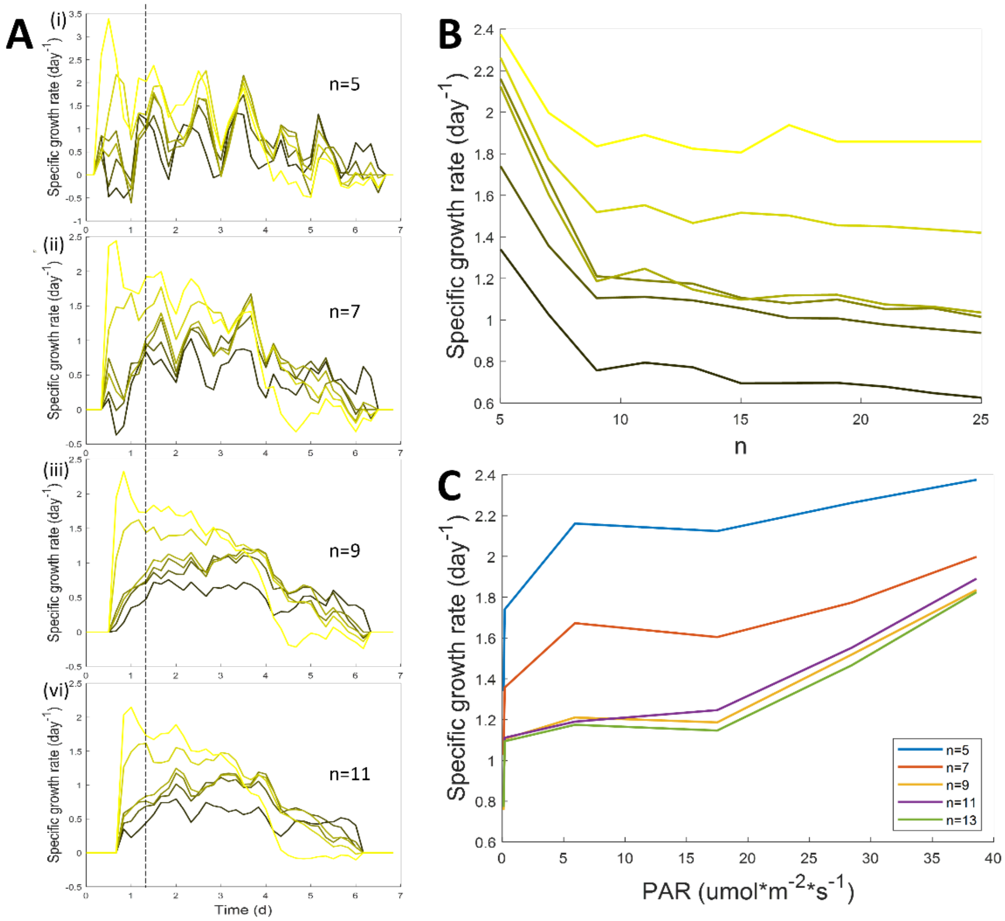

Fig. S2.

Growth rate calculation. (A) Specific growth rates calculated for growth curves in Fig. 2D, by fitting n consecutive data points around each time point to a straight line and taking the slope. Shown here were n=5, 7, 9, and 11. Colors represent the light intensity conditions (as in Fig. 2D). The dashed line marked day 1.3, after which maximum growth rates were determined. **(B)** Maximum specific growth rates after day 1.3 versus n values. Colors represented the light intensity conditions (as in Fig. 2D). **(C)** Maximum specific growth rates after day 1.3 versus light intensities. Colors represented different n values. n=9 was chosen for final data analysis shown in Fig. 4B.

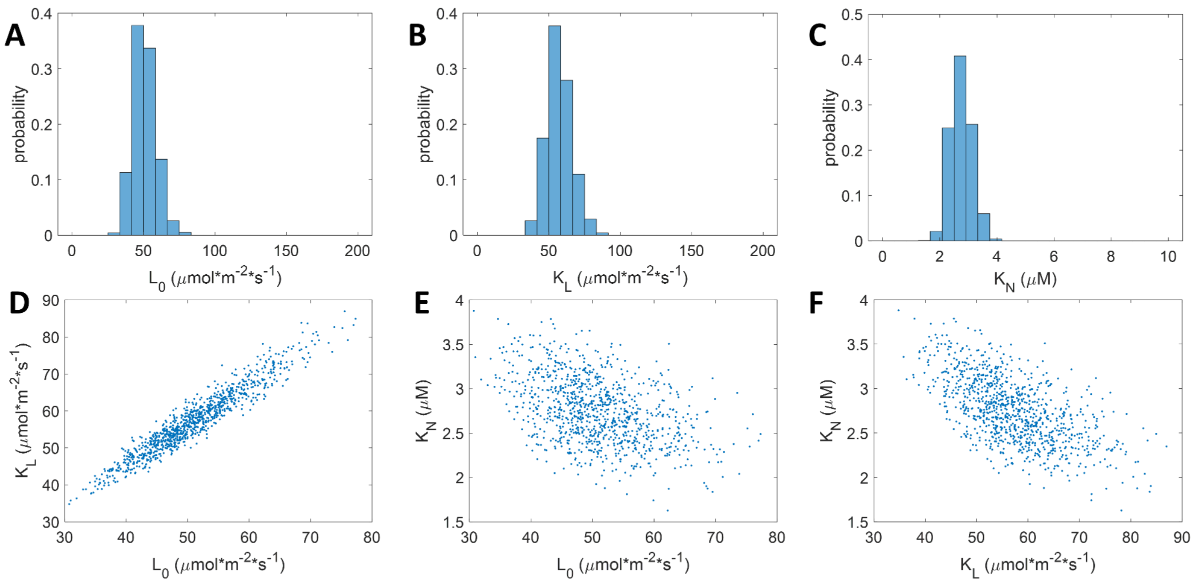

Fig. S3.

Parameters from colimitation model fitting. Distribution of fitted parameters from the general multiplicative model: **(A)** L_0, **(B)** K_L, and **(C)** K_N. Correlation between parameters: **(D-F)**.

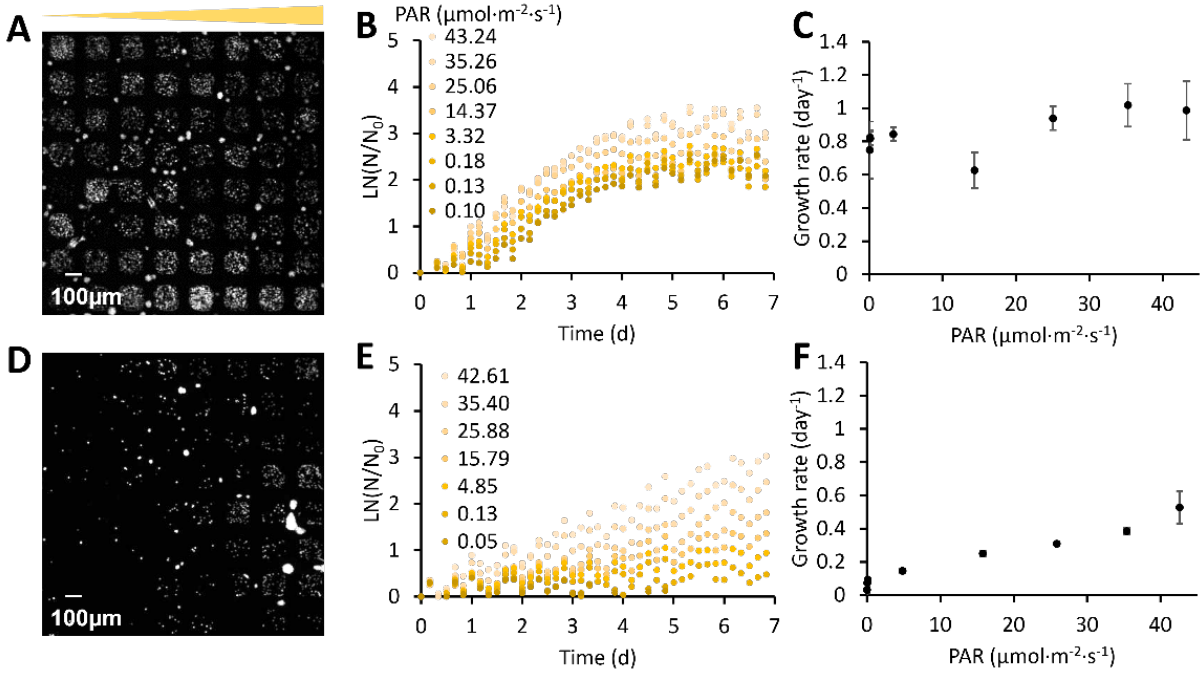

Fig. S4.

Comparing growth of N-starved cells in 10%TAP-N (A-C) and TM-N (D-F). (A) Fluorescence image of algal cells growing in 10% TAP-N under a light gradient on day 7. Contrast was adjusted for illustration purpose. **(B)** Growth curves of algal cells under different light conditions in the columns in (A). **(C)** Growth rate in 10%TAP-N versus light intensity. **(D)** Fluorescence image of algal cells growing in TM-N under a light gradient on day 7. Contrast was adjusted for illustration purpose. **(E)** Growth curves of algal cells under different light conditions in the columns in (A). **(F)** Growth rate in TM-N versus light intensity. Error bars in (C) and (F) represent standard errors of measurements taken on replicate microhabitats under each light intensity condition. 10%TAP-N and TM-N refer to 10%TAP and TM media with no NH4Cl stock solution added (Materials and Methods), and the nitrogen concentration was 5.3µM in both 10%TAP-N and TM-N.

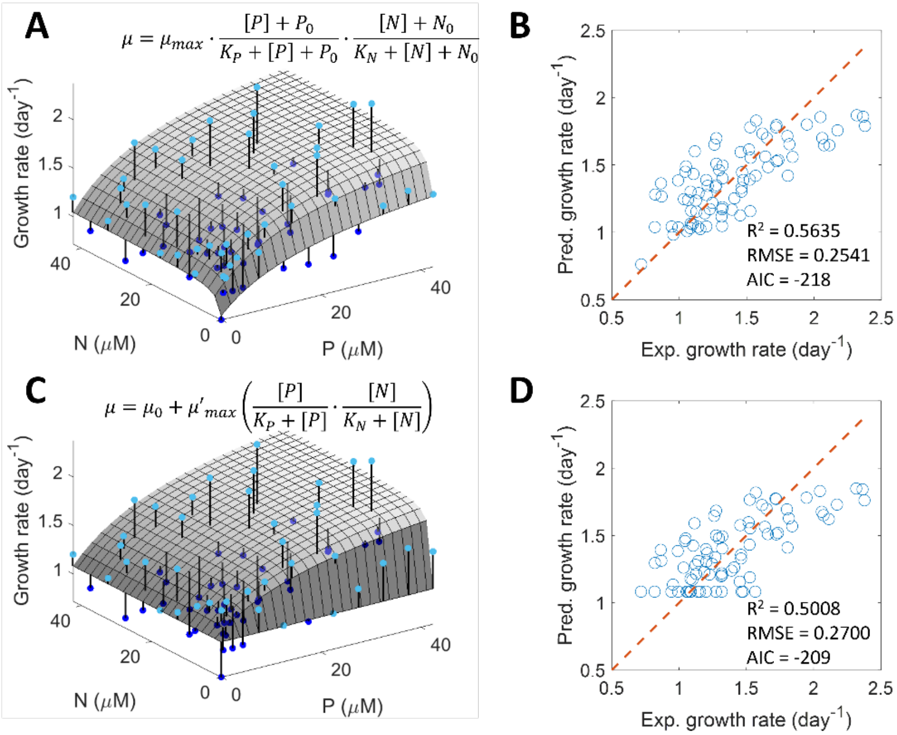

Fig. S5.

Model fitting of phosphorus and nitrogen colimited algal growth. (A,C) The fitted model (surface) and experimental data (dots) from the average of two replicates, resulted from fitting a model considering nutrient storage (equation S1) (A) and one with only baseline growth (C). **(B,D)** The predicted growth rates versus experimental growth rates showing the goodness of fit for results in (A) and (C) respectively.

Movie S1.

(separate file). ***C. reinhardtii*** **cells growth in the array microhabitat for 7 days (0-164 hours) under dual light and nitrogen gradients.** Time laps images were taken using fluorescence imaging at 4-hour intervals. Contrast was adjusted for illustration purposes: the intensity (0-255) corresponds to (0-20000) of the grayscale of original images.

Supplementary References

1. Puzanskiy, R., Romanyuk, D., Shavarda, A., Yemelyanov, V. & Shishova, M. A possible molecular mechanism of Chlamydomonas reinhardtii adaptation to different trophic conditions. *Protistology* **15**, 170-189 (2021).
2. Su, Y. Revisiting carbon, nitrogen, and phosphorus metabolisms in microalgae for wastewater treatment. *Sci Total Environ* **762**, 144590 (2021).
3. Johnson, X. & Alric, J. Central Carbon Metabolism and Electron Transport in Chlamydomonas reinhardtii: Metabolic Constraints for Carbon Partitioning between Oil and Starch. *Eukaryotic Cell* **12**, 776-793 (2013).
4. Chapman, S., Paget, C., Johnson, G. & Schwartz, J.-M. Flux balance analysis reveals acetate metabolism modulates cyclic electron flow and alternative glycolytic pathways in Chlamydomonas reinhardtii. *Frontiers in Plant Science* **6**, 474 (2015).
5. Huppe, H.C. & Turpin, D.H. Integration of Carbon and Nitrogen Metabolism in Plant and Algal Cells. *Annual Review of Plant Physiology and Plant Molecular Biology* **45**, 577-607 (1994).
6. Zhang, X.W., Zhang, Y.M. & Chen, F. Kinetic models for phycocyanin production by high cell density mixotrophic culture of the microalga Spirulina platensis. *Journal of Industrial Microbiology and Biotechnology* **21**, 283-288 (1998).
7. Zhang, X.W., Gong, X.D. & Chen, F. Kinetic models for astaxanthin production by high cell density mixotrophic culture of the microalga Haematococcus pluvialis. *Journal of Industrial Microbiology and Biotechnology* **23**, 691-696 (1999).
8. Bekirogullari, M., Fragkopoulos, I.S., Pittman, J.K. & Theodoropoulos, C. Production of lipid-based fuels and chemicals from microalgae: An integrated experimental and model-based optimization study. *Algal Research* **23**, 78-87 (2017).
9. Yoo, S.J., Kim, J.H. & Lee, J.M. Dynamic modelling of mixotrophic microalgal photobioreactor systems with time-varying yield coefficient for the lipid consumption. *Bioresource Technol* **162**, 228-235 (2014).
10. Adesanya, V.O., Davey, M.P., Scott, S.A. & Smith, A.G. Kinetic modelling of growth and storage molecule production in microalgae under mixotrophic and autotrophic conditions. *Bioresource Technol* **157**, 293-304 (2014).
11. Figueroa-Torres, G.M., Pittman, J.K. & Theodoropoulos, C. Kinetic modelling of starch and lipid formation during mixotrophic, nutrient-limited microalgal growth. *Bioresource Technol* **241**, 868-878 (2017).
12. Liu, F., Yazdani, M., Ahner, B.A. & Wu, M. An array microhabitat device with dual gradients revealed synergistic roles of nitrogen and phosphorous in the growth of microalgae. *Lab Chip* **20**, 798-805 (2020).
13. Poole, H.H. & Atkins, W.R.G. Photo-electric Measurements of Submarine Illumination throughout the Year. *Journal of the Marine Biological Association of the United Kingdom* **16**, 297-324 (1929).

14. Sherwood, B.I. & Gilbert, R.G. On the Universality of the Poole and Atkins Secchi Disk-Light Extinction Equation. *Journal of Applied Ecology* **11**, 399-401 (1974).
